# Comparison of proton therapy and photon therapy for early-stage non-small cell lung cancer: a meta-analysis

**DOI:** 10.1186/s40364-024-00642-5

**Published:** 2024-08-26

**Authors:** Junyi He, Yingxin Liu, Xiaojing Zhang, Butuo Li, Linlin Yang, Haohua Wang, Shijiang Wang, Jinming Yu, Linlin Wang

**Affiliations:** 1 Cheeloo College of Medicine, Shandong University Cancer Center, Shandong University, Jinan, Shandong China; 2 Shandong First Medical University, Shandong Academy of Medical Sciences, Jinan, Shandong China; 3 Department of Radiation Oncology, Shandong Cancer Hospital and Institute, Shandong First Medical University, Shandong Academy of Medical Sciences, Jiyan Road 440, Jinan, 250117 Shandong China

**Keywords:** Proton therapy, Lung cancer, Early stage, Safety, Efficacy, Meta-analysis

## Abstract

The use of proton therapy (PT) in early-stage non-small cell lung cancer (ES-NSCLC) remains controversial, with insufficient evidence to determine its superiority over photon therapy (XRT). We conducted a systematic review of PT trials in ES-NSCLC, analyzing dosimetry, efficacy, and safety across to inform clinical decision-making. Our study showed that PT reduced lung and heart dosimetric parameters compared to XRT, with significant differences in lung V5, lung V10 and mean heart dose (MHD). In terms of efficacy, there were no significant differences in 1-year OS, 3-year OS and 3-year PFS between PT and XRT. For toxicity, no significant difference was observed in treatment-related adverse events (TRAEs) and radiation pneumonitis (RP). Single-arm analysis of PT found that V5, V10, V20 of lung and heart V5 were 13.4%, 11.3%, 7.9% and 0.7%, respectively. The mean lung dose and MHD were 4.15 Gy and 0.17 Gy, respectively. The single-arm pooled 1-, 2-, 3- and 5-year OS rates for PT were 95.3%, 82.5%, 81.3% and 69.3%, respectively. PFS rate and local control rate at 3 years were 68.1% and 91.2%, respectively. The rates of TRAEs of grade ≥ 3 and grade ≥ 2 were 2.8% and 19.8%, respectively. The grade ≥ 2 RP occurred at a rate of 8.7%. In conclusion, PT had acceptable efficacy and safety, and was better at protecting organs at risk than XRT in ES-NSCLC. However, the survival and safety benefit of PT was not significant compared to XRT.

**Supplementary Information:**

The online version contains supplementary material available at 10.1186/s40364-024-00642-5.

*To the editor:* Radiotherapy is now the standard treatment for patients with unresectable early-stage non-small cell lung cancer (ES-NSCLC) [1]. Stereotactic body radiotherapy offers excellent survival outcomes, but is limited by the dose to organs at risk (OARs) [2]. Proton beam, with its Bragg peak, stops precisely at edge of targets, resulting in lower dosimetry of OARs and better dose deposition in tumors. However, some studies have suggested that proton therapy (PT) does not provide a survival benefit over photon therapy (XRT) [3, 4]. Currently, the use of PT in the treatment of ES-NSCLC remains controversial due to insufficient evidence, including the lack of large randomized controlled trials, to definitively establish its superiority over XRT. Therefore, we conducted a systematic review of PT trials in ES-NSCLC, analyzing dosimetry, efficacy, and safety across to inform clinical decisions (Figure S1)0.19 studies were finally included in the meta-analysis, of which 5 were comparative studies [3–6] (Table S1), and 14 were single-arm studies (Table S2).

In terms of dosimetry, PT showed reduced lung and heart dosimetric parameters versus XRT (Fig. 1). For lung, significant reductions were observed in lung V5 with 6.2% (95% confidence interval (CI): 5.0, 7.5) and lung V10 with 2.6% (95% CI: 1.5, 3.6) when compared PT to XRT. No significant difference was found in other parameters of lung, including V20, V40 and mean lung dose (MLD). For the heart, PT was significantly associated with a lower mean heart dose (MHD), with a reduction of 1.4 Gy (95% CI: 0.6, 2.2).

**Fig. 1:**
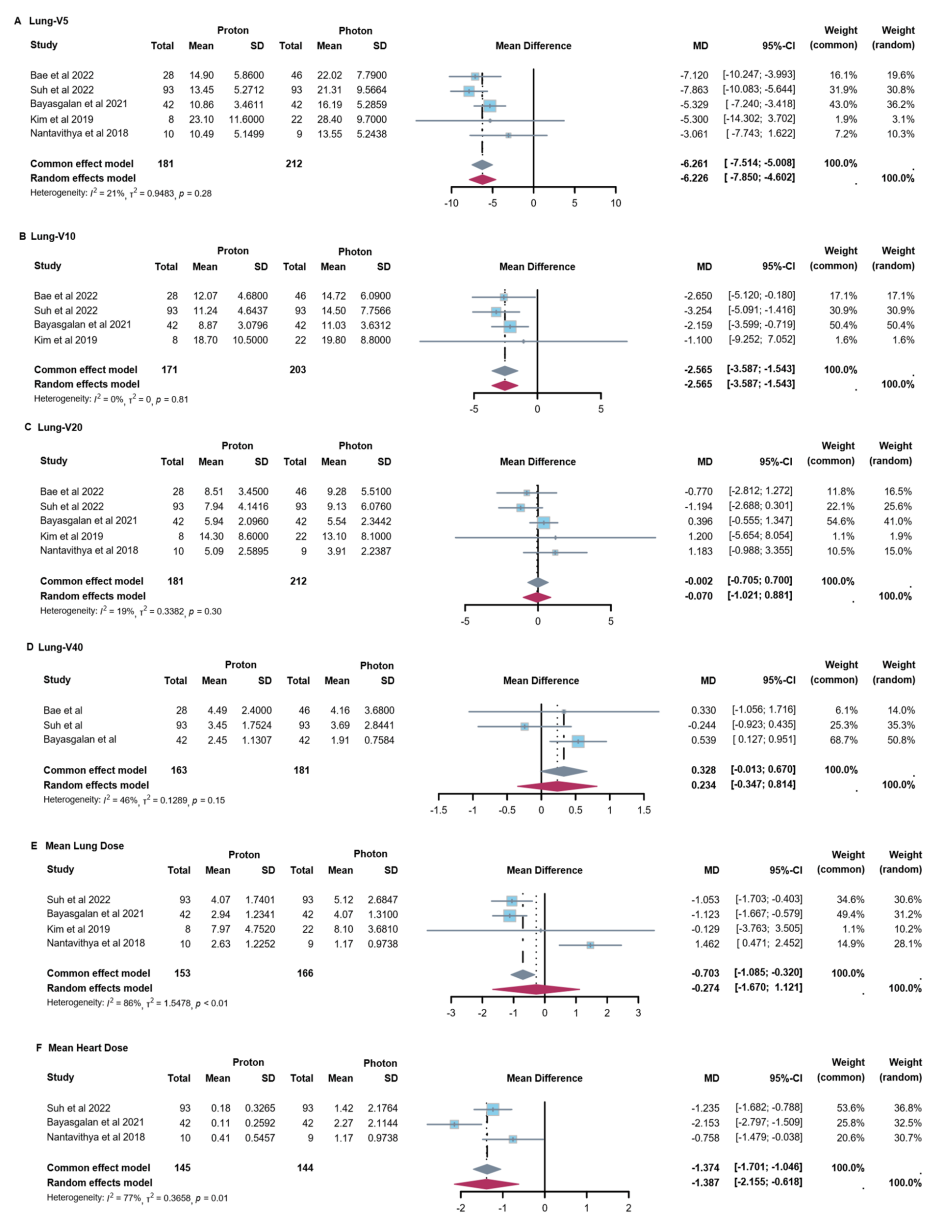
Forest Plots of Dose-Volume Parameters for OARs with Proton vs. Photon Therapy. (**A**) Lung-V5 of PT versus XRT. (**B**) Lung-V10 of PT versus XRT. (**C**) Lung-V20 of PT versus XRT. (**D**) Lung-V40 of PT versus XRT. (**E**) Mean lung dose of PT versus XRT (**F**) Mean heart dose of PT versus XRT

When comparing survival data between PT and XRT, no significant difference was found (Fig. 2A-C). For progression-free survival (PFS), there was a 14.1% (95% CI: -25.0%, 53.2%) increase in 3-year PFS with PT, from 45.5 to 59.6%. For overall survival (OS), there was 1.6% (95% CI: -4.6%, 7.9%) increase in 1-year OS with PT, from 89.7 to 91.3%. While for 3-year OS rate, there was 3.6% (95% CI: -28.6%, 21.5%) decrease with PT, from 59.7 to 56.1%. Regarding treatment-related adverse events (TRAEs), no significant differences were found between PT and XRT in the rates of grade ≥ 2 (OR = 0.58, 95% CI: 0.32, 1.07) and grade ≥ 3 (OR = 0.69, 95% CI: 0.26, 1.79) TRAEs. Similarly, no significant differences were found in the incidence of grade ≥ 2 radiation pneumonitis (RP) (OR = 0.61, 95% CI: 0.29, 1.29) and grade ≥ 3 RP (OR = 0.88, 95% CI: 0.12, 6.51) (Fig. 2D-G).

**Fig. 2:**
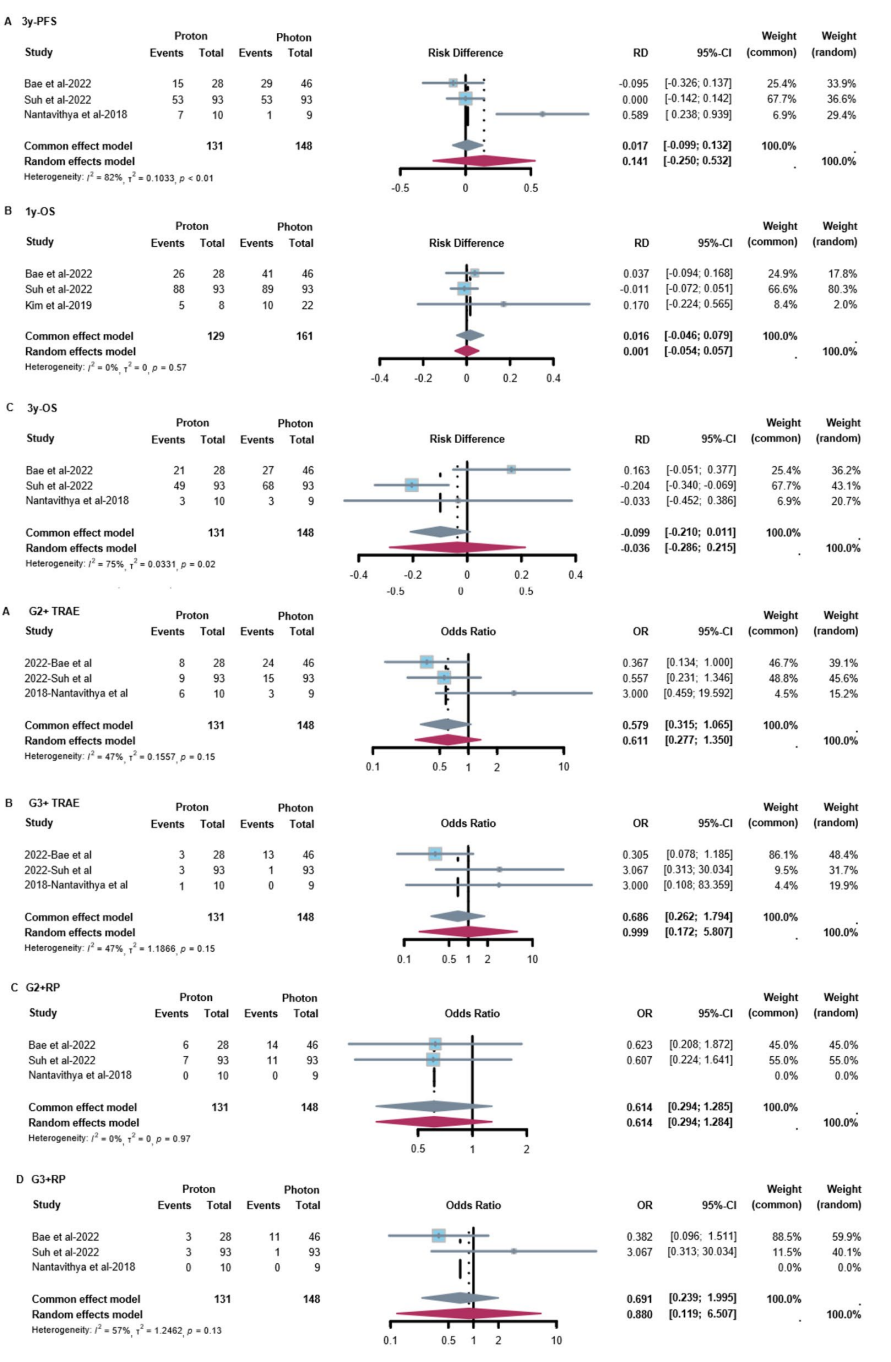
Forest Plots of efficacy and safety with Proton vs. Photon Therapy. (**A**) 3-year PFS of PT versus XRT. (**B**) 1-year OS of PT versus XRT. (**C**) 3-year OS of PT versus XRT. (**D**) G2 + TRAE of PT versus XRT. (**E**) G3 + TRAE of PT versus XRT. (**F**) G2 + RP of PT versus XRT. (**G**) G3 + RP of PT versus XRT

In single-arm analysis of PT, we also assessed dosimetry, efficacy and safety (Figure S2-S4). In terms of dosimetry, pooled V5, V10 and V20 of lung were 13.38% (95%CI: 11.79%, 14.96%), 11.29% (95%CI: 9.00%, 13.59%) and 7.94% (95%CI: 6.32%, 9.56%), respectively. Besides, the pooled MLD was 4.15 Gy (95%CI: 3.04, 5.26). For heart, V5 was 0.69% (95%CI: 0.01%, 1.37%) and the MHD was 0.17 Gy (95%CI: 0.09, 0.24). For esophagus and spinal cord, the maximum dose was 14.26 Gy (95%CI: 1.57, 26.94) and 1.45 Gy (95%CI: 0.58, 2.31), respectively. For survival outcomes of PT, the pooled analysis showed 1-, 2-, 3- and 5-year OS rates were 95.3% (95%CI: 91.8%, 98.8%), 82.5% (95%CI: 77.0%, 87.9%), 81.3% (95%CI: 76.4%, 86.2%) and 69.3% (95%CI: 50.4%, 88.3%), respectively. The 3-year PFS and local control rates were 68.1% (95%CI: 62.1%, 74.0%) and 91.2% (95%CI: 86.9%, 95.5%), respectively. In terms of toxicity, the rates of grade ≥ 3 and grade ≥ 2 TRAEs were 2.8% (95% CI: 1.5%, 4.5%) and 19.8% (95% CI: 15.6%, 25.1%), respectively. The incidences of grade ≥ 2 RP, grade ≥ 2 dermatitis and grade ≥ 2 chest wall pain were 8.7% (95% CI: 5.6%, 11.7%), 7.1% (95% CI: 3.3%, 15.4%) and 3.4% (95% CI: 0%, 6.9%), respectively.

Our meta-analyses showed that PT reduced cardiopulmonary dose but did not differ significantly from XRT in terms of survival outcomes and adverse events. Liao et al. noted a learning curve for PT, suggesting that technological advancements and increased experience improve trial results [7]. Other studies have shown that PT has a more pronounced immunomodulatory effect and causes less lymphopenia than XRT [8–10]. It is reported that XRT combined with immunotherapy can achieve better outcomes for patients [11, 12], suggesting the potential to explore the further benefits of combining PT with immunotherapy.

### Electronic supplementary material

Below is the link to the electronic supplementary material.

Supplementary Material 1

Supplementary Material 2

Supplementary Material 3

Supplementary Material 4

Supplementary Material 5

Supplementary Material 6

Supplementary Material 7

Supplementary Material 8

## Data Availability

No datasets were generated or analysed during the current study.

## Abbreviations

CI: Confidence interval.
ES-NSCLC: Early-stage non-small cell lung cancer
MHD: Mean heart dose
MLD: Mean lung dose
OAR: Organs at risk
OS: Overall survival
PFS: Progression-free survival
PT: Proton therapy
RP: Radiation pneumonitis
TRAEs: Treatment-related adverse events
XRT: Photon therapy
