# Supplementary figures and images for: Comparison of proton therapy and photon therapy for early-stage non-small cell lung cancer: a meta-analysis
Source: Biomark Res. 2024 Aug 26;12:90. doi: 10.1186/s40364-024-00642-5 (PMC11346271; doi:10.1186/s40364-024-00642-5)

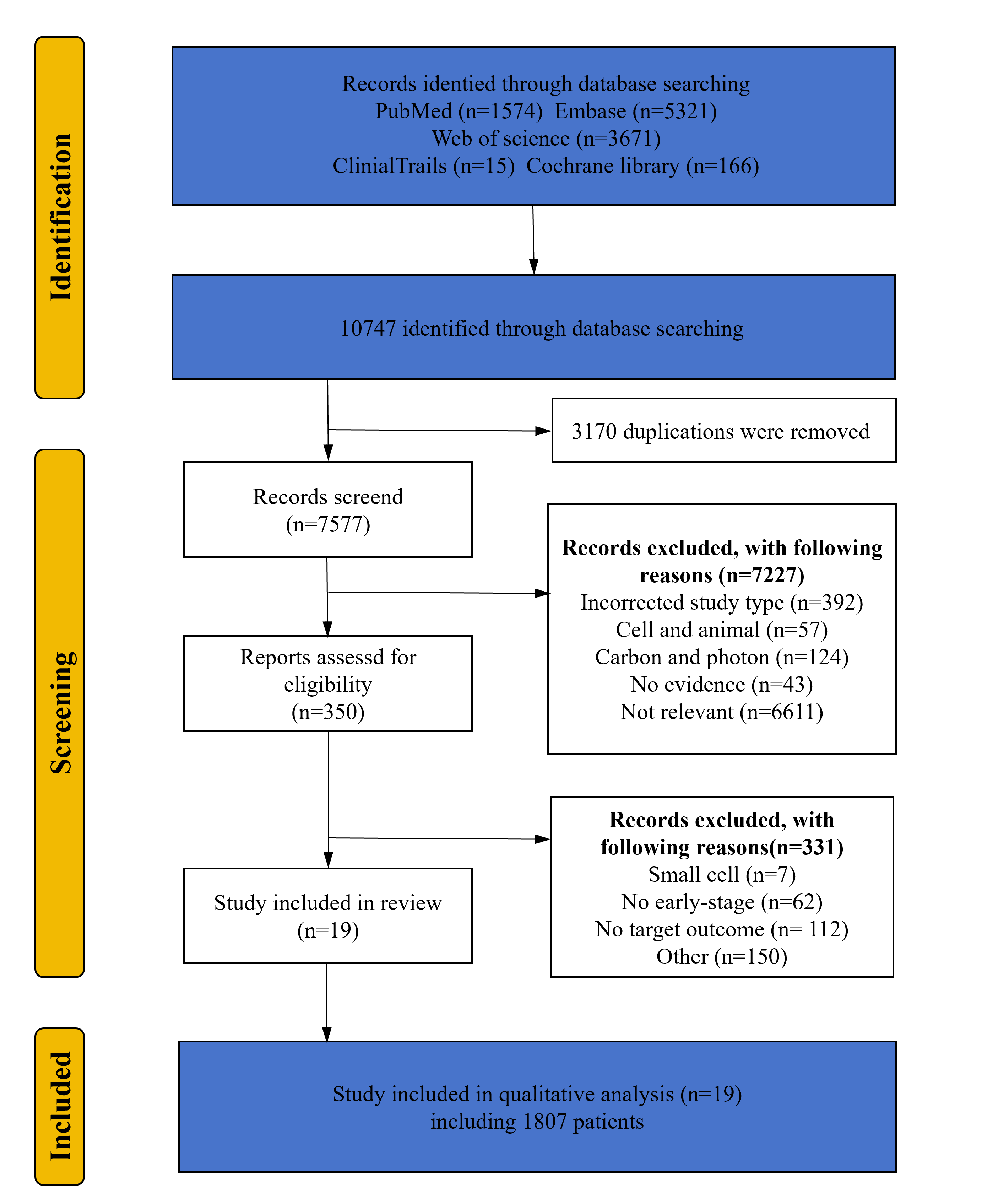

Supplement: Supplementary file 1 — Supplementary Material 1 [file 40364_2024_642_MOESM1_ESM.tif]

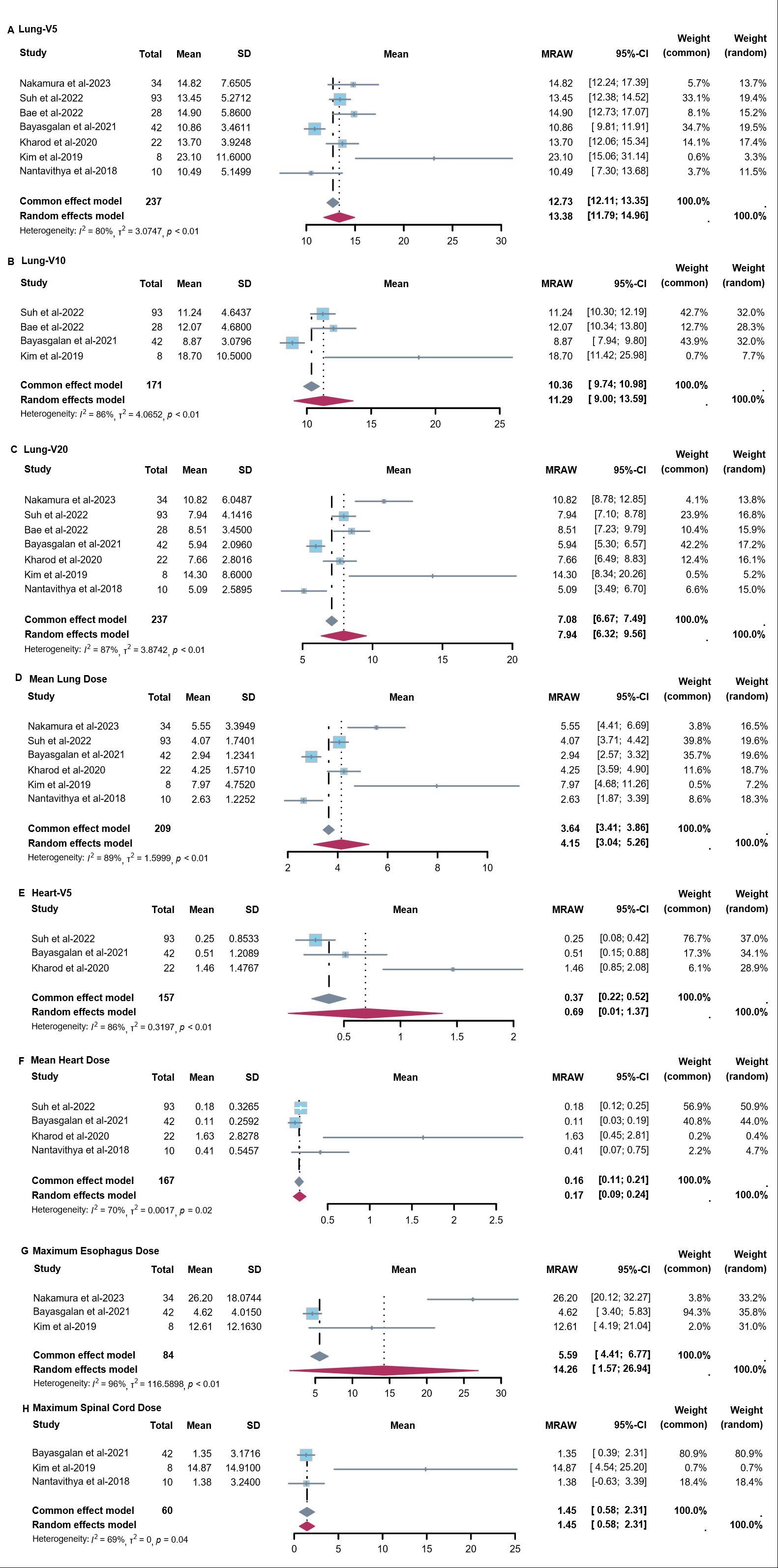

Supplement: Supplementary file 2 — Supplementary Material 2 [file 40364_2024_642_MOESM2_ESM.tif]

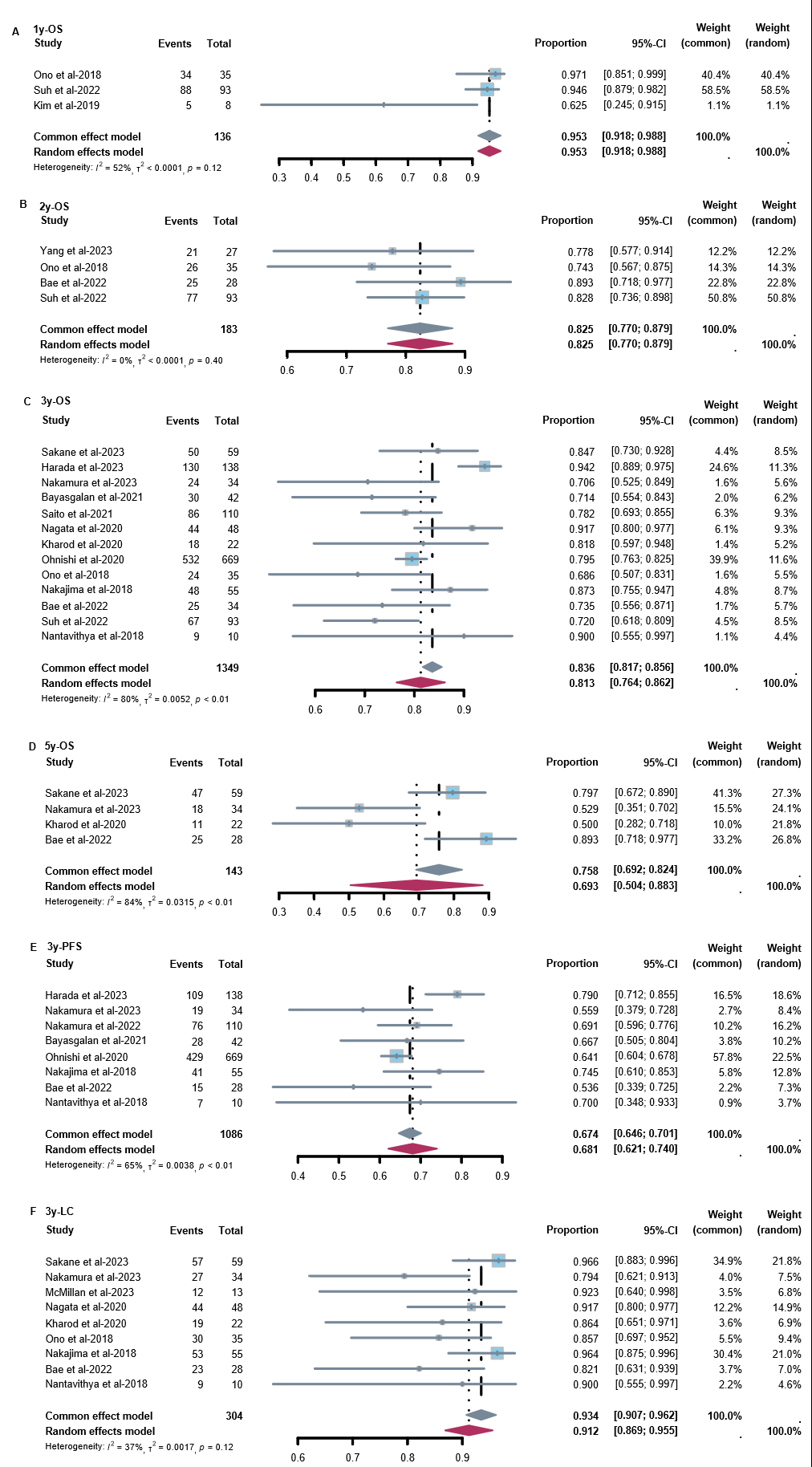

Supplement: Supplementary file 3 — Supplementary Material 3 [file 40364_2024_642_MOESM3_ESM.tif]

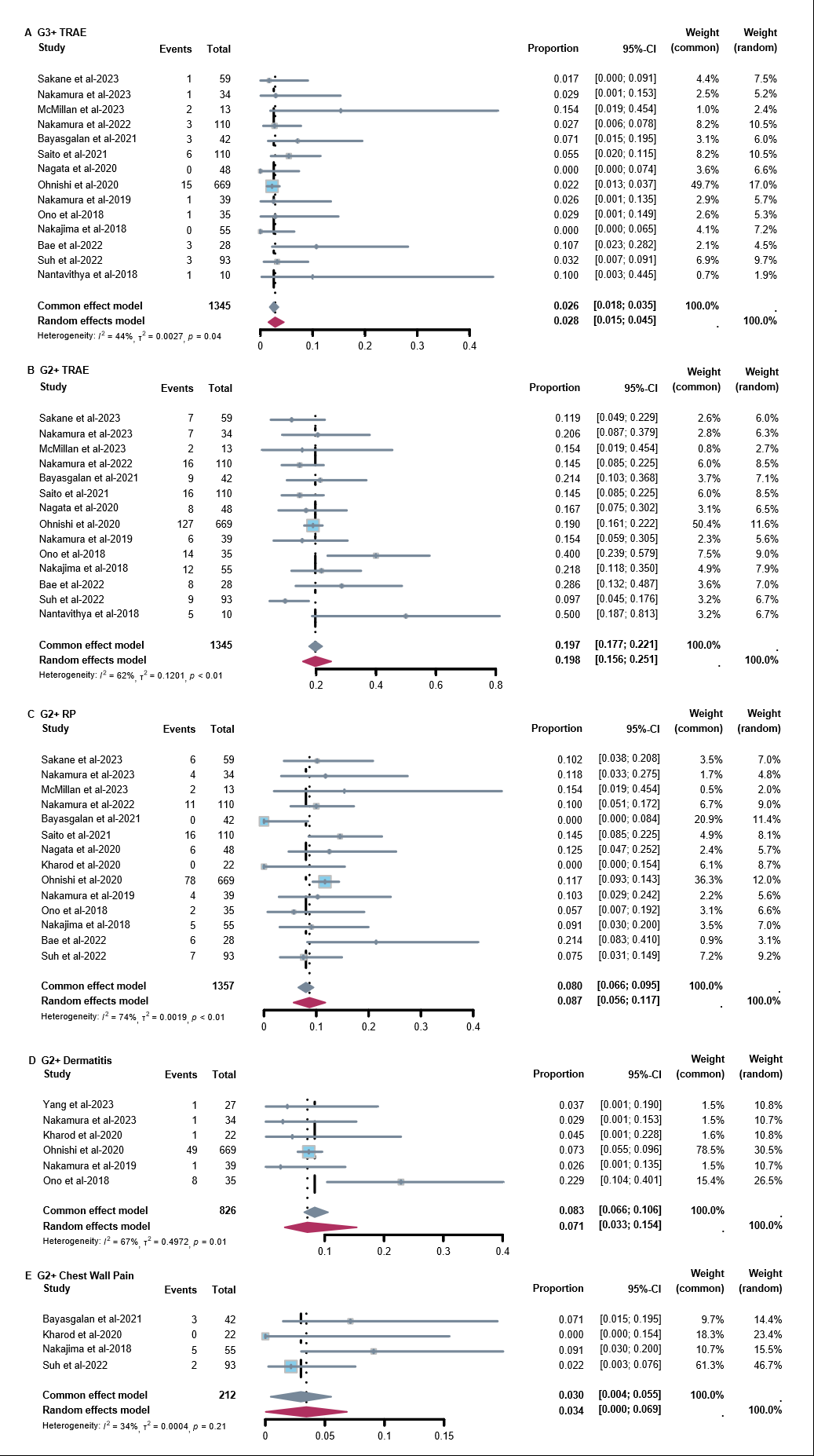

Supplement: Supplementary file 4 — Supplementary Material 4 [file 40364_2024_642_MOESM4_ESM.tif]
