# Supplementary material for: Comparison of proton therapy and photon therapy for early-stage non-small cell lung cancer: a meta-analysis
Source: Biomark Res. 2024 Aug 26;12:90. doi: 10.1186/s40364-024-00642-5 (PMC11346271; doi:10.1186/s40364-024-00642-5)
Supplement: Supplementary file 5 — Supplementary Material 5 [file 40364_2024_642_MOESM5_ESM.docx]

Supplementary Table 1. Description of Prognosis and Toxic Effects After Treatment with Proton or Photon Radiotherapy in Patients with Early-stage Lung Cancer

| Source | Research year range | Follow-up, median (photon vs proton), m | Patients, No. | | Study type | Characteristics (photon vs proton) | | | | | | | Survival (photon vs proton) | Toxic effects, No. (photon vs proton) |
| --- | --- | --- | --- | --- | --- | --- | --- | --- | --- | --- | --- | --- | --- | --- |
|  |  |  | Photon | Proton |  | Age, median, y | T Stage, No. | Pathological type, No. | Operability, No. | Thoracic cite, No. | RT technology, No. | Total radiation dose |  |  |
| Bae et al [25] | 2010 - 2019 | 32 vs 26 | 46 | 28 | RO | 75 (70–78) vs 73 (68–76) | T1 38 vs 22  T2 8 vs 6 | SCC 12 vs 8  AD 16 vs 10  Other 3 vs 0 Unknown 15 vs 10 | 13 vs 0 | LLL 5 vs 7 LUL 12 vs 6 RLL 11 vs 8  RML 3 vs 1  RUL 15 vs 6 | 3D-CRT /IMRT (29/17) vs PSPT/IMPT (4/24) | 60 Gy/4 f | OS: 2-y 81.3% vs 74.5% 5-y 43.4% vs 74.5% PFS: 2-y 67.7% vs 61.9%  5-y 62.9% vs 55.0% LC: 2-y 94.9% vs 91.3%  5-y 89.6% vs 81.1% | G3+RP 11 vs 3, G3+Musculoskeletal 1 vs 0, G3+ Skin 1 vs 0 G2+RP 14 vs 6, G2+Musculoskeletal 7 vs 2, G2+ Skin 3 vs 0 |
| Suh et al  [26] | 2015 - 2019 | 27 vs 27 | 93 | 93 | RO | 74 (69-80) vs 75 (69-80) | T2a 29 vs 30 Other 64 vs 63 | AD 28 vs 31 Other 65 vs 62 | 0 vs 0 | Peripheral 62 vs 60 Central 31 vs 33 | XRT vs PT | 48-70 Gy/ 4-22 f | OS: 1-y 96.2% vs 94.5% 2-y 89.2% vs 83.2% 3-y 73.3% vs 72.5% PFS: 1-y 82.7% vs 82.7% 2-y 73.4% vs 65.4% 3-y 57.0% vs 57.3% | G3+RP 1 vs 3 G2+RP 11 vs 7, G2+Chest wall pain 3 vs 2, G2+Rib fracture 1 vs 0 |
| Bayasgalan et al  [27] | 2016 - 2019 | NA | 42 | 42 | RO | 78 (58-92) vs 78 (58-92） | T1a 16 vs 16 T1b 17 vs 17 T2a 9 vs 9 | SCC 9 vs 9 AD 21 vs 21 Other 3 vs 3 Unknown 9 vs 9 | NA | Peripheral 9  Close to chest wall 22 Central 11 | VMAT vs PT | 50-70 CGE/4-10 f | NA | NA |
| Kim et al  [28] | 2010 - 2017 | 11 vs 11 | 22 | 8 | RO | 75 (55–84) vs 77 (62–85) | T1a-T2a 15 vs 4  T2b-T3 7 vs 4 | SCC 10 vs 5  AD 5 vs 2  Other 1 vs 0  Unknown 6 vs 1 | NA | NA | SBRT/3D-CRT/IMRT (11/10/1) vs SBPT/IMPT (6/2) | 60 Gy/4-20 f vs 60-64 Gy/4-20 f | OS: 1-y 46.4% vs 66.7% | Treatment-related death 4 vs 0 severe treatment-related pulmonary complications 9 vs 1 |
| Source | Research year range | Follow-up, median (photon vs proton), m | Patients, No.  Study type | | Study type | Characteristics (photon vs proton) | | | | | | | Survival (photon vs proton) | Toxic effects, No. (photon vs proton) |
|  |  |  | Photon | Proton |  | Age, median, y | T Stage, No. | Pathological type, No. | Operability, No. | Thoracic cite, No. | RT technology, No. | Total radiation dose |  |  |
| Nantavithya et al  [29] | 2012 - 2014 | 27 vs 37 | 9 | 10 | PO | 76 (66-88) vs 72 (53-87) | T1 6 vs 5 T2 1 vs 2 T3 0 Vs 1 Other 2 vs 2 | SCC 3 vs 4  AD 6 vs 6 | 0 vs 0 | Peripheral 0 vs 0 Central 9 vs 10 | SBRT vs SBPT | 50 Gy/4 f | OS: 3-y 27.8% vs 90% PFS: 3-y 11.1% vs 70% LC: 3-y 87.5% vs 90% | G3+RP 0 vs 0, G3+ Skin fibrosis 0 vs 1 G2+dyspnoea 1 vs 3, G2+Cough 0 vs 1, G2+ Fatigue 2 vs 1 |
