# Supplementary material for: Comparison of proton therapy and photon therapy for early-stage non-small cell lung cancer: a meta-analysis
Source: Biomark Res. 2024 Aug 26;12:90. doi: 10.1186/s40364-024-00642-5 (PMC11346271; doi:10.1186/s40364-024-00642-5)
Supplement: Supplementary file 6 — Supplementary Material 6 [file 40364_2024_642_MOESM6_ESM.docx]

Supplementary Table2. Description of Prognosis and Toxic Effects for Proton Therapy in Patients with Early-stage Lung Cancer

| Source | Year | Patients, No. | Study type | Research year range | FU, median, m | RT technique | Operability, No. | Age(range), y | T stage, No. | Pathological type, No. | Total dose | Survival | Grade2 toxicity | Grade3 toxicity | Grade4-5 toxicity |
| --- | --- | --- | --- | --- | --- | --- | --- | --- | --- | --- | --- | --- | --- | --- | --- |
| Sakane et al | 2023 | 59 | RO | 2013.07 - 2020.12 | 58 (9-106) | PSPT | 41 | 72 (66-76) | T1mi/T1a 8  T1b 20  T1c 20  T2a 11 | AD 44  SCC 12  Other 3 | 72.6 Gy/22 f (central)  66 Gy/10 f (peripheral) | 3y-OS 84.1%, 5y-OS 79.1% 3y-LC 96.6% | RP 6 | RP 1 | 0 |
| Yang et al | 2023 | 27 | PO | 2018.03 -2020.08 | 29 (1-62) | PT | 0 | 74 (56-84) | T1 14 T2 9 T3 4 | AD 10  SCC 6  Other 11 | 64 CGE/8 f | 2y-OS 76.5% 2-y LC 73.5% | NA | RP 1, Dermatitis 1 | NA |
| Harada et al | 2023 | 138 | PO | NA | 43 (37-49) | PT | 138 | NA | T1-T2a | NA | NA | 3y-OS 93.9%  3y-PFS 79.0% | NA | 0 | NA |
| Nakamura et al | 2023 | 34 | RO | 2006 -2019 | 51 (6-100) | PSPT | 11 | 77 (55-88) | T1a 12 T1b 6 T2a 8 T2b 5 T3 2 T4 1 | AD 14 SCC 9  Other 11 | 72.6 CGE/22 f 75 CGE/25 f | 3y-OS 70.4%, 5y-OS 51.7% 3y-LC 80.5%, 5y-LC 70.0% 3y-PFS 55.5% ,5y-PFS 39.2% | Acute: Dermatitis 1, Esophagitis 1 Late: RP 3, Lung infection 1 | RP 1 | 0 |
| McMillan et al | 2023 | 13 | RO | 2019.12- 2022.11 | 11 | IMPT | 2 | 75 (65-83) | NA | AD 3  SCC 6  Other 4 | 50 CGE/10 f | 3y-LC 89% | 0 | RP 1 | RP 1 |
| Nakamura et al | 2022 | 110 | RO | 2009 - 2019 | 37 (5-131) | PSPT | 40 | 77 (53-89) | Tis 1  T1a 7  T1b 39  T1c 26  T2a 19  T2b 11 T3 7 | AD 34 SCC 20  NOS 4  Other 52 | 66 CGE/10 f 72.6 CGE /22 f 70 CGE /25 f 80 CGE /20 f | 3y-OS 80% 3y-PFS 69% | RP 8, Rib fracture 4, Skin ulceration 1 | RP 3 | 0 |
| Bayasgalan et al | 2021 | 42 | RO | 2016.01-2019.12 | 40 (32–48) | PSPT | 39 | 78 (58–92) | T1a 16  T1b 17  T2a 9 | AD 21  SCC 9  Other12 | 60 CGE / 4f  50 CGE / 4f  70 CGE / 10f  60 CGE / 10f | 3y-OS 71.8% 3y-PFS 66.9% | Chest wall pain 3, Cough 3 | Dyspnea 3 | 0 |
| Saito et al | 2021 | 110 | RO | 2002 - 2017 | 38(2-177) | PSPT | 56 | 76 (52-88) | T1a 38 T1b 35 T2a 33 T2b 4 | AD 44 SCC 24  NOS 6  Unknown 36 | 66 Gy/10 f 72.6 Gy/22 f | 3y-OS 78.2% | RP 10 | RP 6 | 0 |
| Nagata et al | 2020 | 48 | RO | 2011.04- 2015.09 | 49 | PT | 10 | 70.9 (9.2) | NA | NA | 66 CGE/10 f | 3y-OS 91.7% 3y-OS 92.5% | RP 6 Cough 2 | 0 | 0 |

| Source | Year | Patients, No. | Study type | Research year range | FU, median, m | RT technique | Operability, No. | Age(range), y | T stage, No. | Pathological type, No. | Total dose | Survival | Grade2 toxicity | Grade3 toxicity | Grade4-5 toxicity |
| --- | --- | --- | --- | --- | --- | --- | --- | --- | --- | --- | --- | --- | --- | --- | --- |
| Kharod et al | 2020 | 22 | PO | 2009 - 2018 | 42 (2-106) | PT | 0 | 72 (58–90) | T1a 2  T1b 3  T1c 5  T2b 1  T2a 11 | AD 8  SCC 12 Other 2 | 60 Gy/10 f (central)  48 Gy/4 f (peripheral) | 3y-OS 81%, 5y-OS 49% 3y-LC 86% | Acute: Hypoxia2 Dyspnea 1 Cough 1 Radiation Dermatitis 1 Late: Hypoxia 3 Dyspnea 2 Bronchial stricture 1 Pleural effusions 2 Weight Loss 4 | Acute: Hypoxia 1 Late:  Hypoxia 2, Bronchial stricture 1 | 0 |
| Ohnishi et al | 2020 | 669 | RO | 2004.01 - 2013.12 | 38 (1-155) | PSPT | 351 | 76 (42-94) | T1a 265 T1b 216 T2a 201 | NA | NA | 3y-OS 79.5% 3y-PFS 64.1% | RP 66 Dermatitis 46 | RP 7, Dermatitis 3 | RP 5 |
| Nakamura et al | 2019 | 39 | RO | 1999 - 2015 | 48 (4–140) | PT | 28 | 75 (48–88) | T1 21 T2 18 | AD 16  SCC 8 Other 15 | 60 Gy/20 f  70 Gy/20 f  60 Gy/10 f  75Gy/25 f  66 Gy/10 f  80 Gy/20 f  88 Gy/20 f | 2y-OS 97.4%  2y-PFS 71.8% | Acute: dermatitis 1  Late: RP 4 | Dyspnoea 1 | 0 |
| Ono et al | 2018 | 35 | RO | 2009 - 2015 | 34 (10–72) | PSPT | 10 | 82 (80–87) | T1 13  T2 13  T3 8 T4 1 | AD 17  SCC 17 Other 1 | 80 Gy/25 f  66 Gy/10 f | 1y-OS 97.1%, 2y-OS 74.3%, 3y-OS 67.2%  3y-LC 86.5% | RP 2, Rib fracture 4, Dermatitis 7 | Dermatitis 1 | 0 |
| Nakajima et al | 2018 | 55 | PO | 2013.07 - 2017.02 | 35 (12–54) | PSPT | 34 | 71 (48–88） | NA | AD 44  SCC 10 Other 1 | 72.6 Gy/22 f (central) 66 Gy/10 f (periphery) | 3y-OS 87%  3y-LC 96%  3y-PFS 74% | RP 5,  Rib fracture 2, Chest wall pain 5 | 0 | 0 |
|  | | | | | | | | | | | | | | | |
