# Supplementary material for: Comparison of proton therapy and photon therapy for early-stage non-small cell lung cancer: a meta-analysis
Source: Biomark Res. 2024 Aug 26;12:90. doi: 10.1186/s40364-024-00642-5 (PMC11346271; doi:10.1186/s40364-024-00642-5)
Supplement: Supplementary file 7 — Supplementary Material 7 [file 40364_2024_642_MOESM7_ESM.docx]

**Figure legends**

Table S1. Abbreviations: RO, retrospective; PO, prospective; AD, adenocarcinoma; SCC, squamous cell carcinoma; PT, proton therapy; XRT, photon therapy; LLL, left lower lobe; LUL, left upper lobe; RLL, right lower lobe; RML, right middle lobe; RUL, right upper lobe; 3D-CRT, 3D conformal radiotherapy; VMAT, volumetric modulated arc therapy; IMRT, intensity modulated radiation therapy; SBRT: stereotactic body radiation therapy; SBPT: stereotactic body proton therapy; IMPT, intensity modulated proton therapy; Gy, Gray; CGE, cobalt Gray equivalent; f, fraction; NA, not available or not applicable; OS, overall survival; PFS, progression-free survival; LC, local control; RP: radiation pneumonitis; G2+: grade 2 or higher; G3+: grade 3 or higher.

Table S2. Abbreviations: RO, retrospective; PO, prospective; FU, follow-up; AD, adenocarcinoma; SCC, squamous cell carcinoma; NOS: not otherwise specified; f, fraction; PSPT, passive scattering proton therapy; PT, proton therapy; IMPT, intensity modulated proton therapy; Gy, Gray; CGE, cobalt Gray equivalent; f, fraction; NA, not available or not applicable; OS, overall survival; PFS, progression-free survival; LC, local control; RP: radiation pneumonitis.

Figure S1. Flowchart of the Study Screening and Evaluation Process.

Figure S2. Forest Plots of Dose-Volume Parameters for OARs with Proton Therapy. A) Lung-V5 of proton therapy. B) Lung-V10 of proton therapy. C) Lung-V20 of proton therapy. D) Mean lung dose of proton therapy. E) Heart-V5 of proton therapy. F) Mean heart dose of proton therapy. G) Maximum esophagus dose of proton therapy. H) Maximum spinal cord dose of proton therapy.

Abbreviations: OARs organs at risk; SD standard deviation; MRAW mean raw; CI confidence interval.

Figure S3. Forest Plots of survival for Proton Therapy. A) 1-year OS rate for proton therapy. B) 2-year OS rate for proton therapy. C) 3-year OS rate for proton therapy. D) 5-year OS rate for proton therapy. E) 3-year PFS rate for proton therapy. F) 3-year LC rate for proton therapy.

Abbreviations: y year; OS overall survival; PFS progression-free survival; LC local control; CI confidence interval.

Figure S4. Forest Plots of Toxic Effects for Proton Therapy. A) G3+ TRAE rate for proton therapy. B) G2+ TRAE rate for proton therapy. C) G2+ RP rate for proton therapy. D) G2+ dermatitis rate for proton therapy. E) G2+ chest wall pain rate for proton therapy.

Abbreviations: TRAE treatment-related adverse events; G2+ grade 2 or higher; G3+ grade 3 or higher; RP radiation pneumonitis; CI confidence interval.
