# Supplementary material for: Comparison of proton therapy and photon therapy for early-stage non-small cell lung cancer: a meta-analysis
Source: Biomark Res. 2024 Aug 26;12:90. doi: 10.1186/s40364-024-00642-5 (PMC11346271; doi:10.1186/s40364-024-00642-5)
Supplement: Supplementary file 8 — Supplementary Material 8 [file 40364_2024_642_MOESM8_ESM.docx]

**Supplementary Methods**

**Registration**

This meta-analysis followed the Preferred Reporting Items for Systematic Reviews and Meta-Analyses (PRISMA) reporting guideline. This study has been prospectively registered at International prospective register of systematic reviews (PROSPERO) and the registration ID is CRD42024526511.

**Search strategy**

We searched PubMed, Embase, Cochrane, Web of Science, and ClinicalTrials for clinical trials of PT in lung cancer, using a combination of subject and free text terms to identify eligible studies published before 14 December 2023. The following MeSH and free words were used in the searches: ((Lung Neoplasms[Mesh]) OR (Pulmonary Neoplasms) OR (Neoplasms, Lung) OR (Lung Neoplasm) OR (Neoplasm, Lung) OR (Neoplasms, Pulmonary) OR (Neoplasm, Pulmonary) OR (Pulmonary Neoplasm) OR (Lung Cancer) OR (Cancer, Lung) OR (Cancers, Lung) OR (Lung Cancers) OR (Pulmonary Cancer) OR (Cancer, Pulmonary) OR (Cancers, Pulmonary) OR (Pulmonary Cancers) OR (Cancer of the Lung) OR (Cancer of Lung)) AND (("Proton Therapy"[Mesh] ) OR (Proton Therapies) OR (Therapies, Proton) OR (Therapy, Proton) OR (Proton Beam Therapy) OR (Proton Beam Therapies) OR (Therapies, Proton Beam) OR (Therapy, Proton Beam) OR (Proton Beam Radiation Therapy)). The selection process and the reasons for excluding studies is describes in Supplementary Figure 1.

**Statistical analyses**

For continuous variables, means and standard deviations were combined. If the study described a median and range for continuous variables, the mean was estimated using the method proposed by Luo et al. [1] and estimated standard deviation of the sample was from Wan et al. [2]. For ratio variables, literature median rate values were pooled, and 95% confidence intervals (CIs) were computed using metaprop command analysis. Where the distribution type of individual ratios was not normal, they were transformed with methods such as double inverse sine transformation to enhance the trustworthiness of the combined outcomes. In cases where partial survival rates were not given but Kaplan-Meier survival curves were provided, we used WebPlotDigitizer 4.7 obtain estimated survival rates from the curves. The I^2^-statistic was used to assess statistical heterogeneity among different studies. A fixed-effects model was used if there was no significant heterogeneity among studies (I^2^ <50%); otherwise, a random-effects model was employed. In the case of single-rate analyses where there was large heterogeneity, the random effects model was used regardless of whether I^2^ was greater than 50%. All analyses were subjected to meta-analysis using the R 4.1.2 with “meta” packages.

**References**

1. Luo D, Wan X, Liu J, Tong T. Optimally estimating the sample mean from the sample size, median, mid-range, and/or mid-quartile range. Stat Methods Med Res. 2018;27:1785–805.

2. Wan X, Wang W, Liu J, Tong T. Estimating the sample mean and standard deviation from the sample size, median, range and/or interquartile range. BMC Med Res Methodol. 2014;14:135.
